# Fragile X-Associated Disorders in Serbia: Baseline Quantitative and Qualitative Survey of Knowledge, Attitudes and Practices Among Medical Professionals

**DOI:** 10.3389/fnins.2018.00652

**Published:** 2018-09-21

**Authors:** Dejan B. Budimirovic, Smiljana Cvjetkovic, Zoran Bukumiric, Phan Q. Duy, Dragana Protic

**Affiliations:** ^1^Clinical Trials Unit, Fragile X Clinic, Department of Psychiatry, Clinical Research Center, Kennedy Krieger Institute, Johns Hopkins Medical Institutions Baltimore, MD, United States; ^2^School of Medicine, Belgrade University, Belgrade, Serbia; ^3^Krieger School of Arts & Sciences, Johns Hopkins University, Baltimore, MD, United States

**Keywords:** KAP survey, fragile X-associated disorders, fragile X premutation, Serbia, genetics

## Abstract

We conducted a knowledge, attitude, and practice (KAP) survey of fragile X-associated disorders (FXD) in Serbia in order to obtain baseline quantitative and qualitative KAP data on fragile X mental retardation 1 gene (*FMR1*) pre- and full mutations (PM, FM). The survey's 16-item questionnaire included a knowledge component (12/16), such as self-assessment knowledge (SAK) and factual knowledge (FK, 2/5 questions for PM, FXTAS and FXPOI). Education-directed attitudes in the FXD field and *FMR1 DNA* testing practices had 4/16 items, including brief case vignettes of FXTAS and FXPOI, respectively. The study's cohort consisted of primary care physicians (referred to as “physicians” in the rest of the text) throughout Serbia (*n* = 284, aged 26–64 years, 176/284, 62.2% in Belgrade, Serbia) and senior medical students (*n* = 245, aged 23–30 years; 33.5% males) at the Belgrade School of Medicine. Strikingly, half of the survey respondents indicated “not having any” knowledge for the fragile X gene premutation and FXD. Physicians were more likely to indicate “not having any” knowledge than students (41.2% of physicians vs. 13.1% of students, *P* < 0.05). Roughly half of the students had “minimal knowledge” (53.5 vs. 30.5% of physicians, *P* < 0.05). Low FK was common in the cohort, as few physicians had “all correct answers” (7.5 vs. 3.7% of students, *P* < 0.05; 16.5 vs. 9.5% of students for the 2/5 premutation-related questions). Statistical analyses identified physicians' practice setting and length of clinical experience as predictors of the lack of FK on questions related to FXD. Physicians were more likely than students to indicate “strongly agreed” to expand their knowledge of the gene premutation and FXD (90.9 vs. 66.7% of students, *P* < 0.01). However, students more frequently indicated that they are willing to recommend DNA testing in their future practices than physicians (93.5 vs. 64.8% of physicians, *P* < 0.001). In *conclusion*, there is a major gap in knowledge regarding fragile X gene PM and FXD among the study's participants in Serbia. The study's informative-educational survey serves as an initial step in the process of enhancing the KAP of medical professionals with regards to the fragile X gene premutation and FXD.

## Introduction

Knowledge, attitude, and practice (KAP) study is one of the most popular and widely used cross-sectional methods. Since 1962, this quantitative approach has revealed qualitative and quantitative information that can be used to understand misconceptions and confusion in a region of interest (World Health Organization, 2008). Such issues represent hurdles to the implementation of desired policies in that particular area. Data collected from KAP studies may provide essential information needed to make strategic decisions, organize educational program, and estimate resources required for various activities in specific fields (World Health Organization, 2008). An increasing number of papers related to KAP has been published in the health field over the last 55 years (2 articles in 1962 and 1 in 1963. vs. 7,013 in 2015), demonstrating a growing interest in and the importance of KAP studies (Torres-Vallejo et al., 2013).

KAP studies capturing disorders under the umbrella of fragile X-associated disorders (FXD), including fragile X syndrome (FXS), surveyed mostly medical professionals in the U. S. about fragile X knowledge, including fragile X screening (Acharya and Ross, 2009; Kemper and Bailey, 2009; Acharya and Schindler, 2013). Kemper and Bailey (2009) found that many of pediatricians surveyed from 17 U. S. states did not have sufficient knowledge to discuss FXS with family members of at-risk children who exhibit developmental delay. Similar findings were found in KAP studies conducted internationally. For example, senior medical school students in China were surveyed for their basic knowledge of FXD, population-based screening, confidentiality, and reproductive options for the fragile X gene mutation carriers (Li et al., 2013). Li et al. (2013) demonstrated that only less than one third of the participants have heard about FXD (Li et al., 2013). Together, these studies found a major gap in FXD knowledge among the study's participants.

Expansion of CGG triplet repeats in the fragile X mental retardation 1 (*FMR1*) gene causes genetic diseases under the umbrella of FXD. A full mutation (FM, ≥200 CGG repeats) of the fragile X gene results in FXS, which is often expanded from the premutation in carriers (PM, 55-200 CGG repeats) (Hagerman et al., 2009). In the general population, the rate of PM is found in approximately 1:130–1:250 females and in 1:250–1:810 males (Tassone et al., 2014), which is 10 times higher than the rate of the FM. It is noteworthy that the prevalence of premutation is highest in Columbia and Israel (1:100 females) and lowest in Japan (1:1,674 females) (Man et al., 2017). Clinical manifestations of the premutation include fragile-X-associated tremor/ataxia syndrome (FXTAS) in adult males (Hagerman and Hagerman, 2013), fragile-X-associated primary ovarian insufficiency (FXPOI), and the fragile-X-associated diminished ovarian reserve (FXDOR) in adult females (Hagerman et al., 2001; Man et al., 2017). Furthermore, results of recent published studies linked many other clinically relevant medical (Winarni et al., 2012; Au et al., 2013) and psychiatric (Roberts et al., 2009) manifestations with the PM, including autism spectrum disorder (ASD) (Farzin et al., 2006). Yet, much effort remains to specify these disorders as well as to disentangle their molecular mechanisms, including genomic (copy number variants) studies (Lozano et al., 2014), which will explain the phenotypic variability. Thus, the clinical impact of a PM is potentially enormous worldwide, including the possibility of expansion into a FM in the next generation and development of the FXS as the most common cause of inherited intellectual disability (ID) in males and the leading single-gene mutation associated with ASD (Hagerman et al., 2009; Budimirovic and Kaufmann, 2011; Kaufmann et al., 2017). Despite the importance of the spectrum of clinical disorders associated with the PM and the high prevalence rate, relatively little attention has been paid to it. For example, there is an overall lack of cognizance among clinicians regarding the distinction between the PM disorders and FXS (Tassone et al., 2014). Therefore, testing for mutations of the fragile X gene, preconception genetic counseling for carriers of the PM, and bringing together professionals with different expertise in the field as well as disseminating knowledge about premutation through publications are of much importance (Tassone et al., 2014).

KAP surveys are relatively easy to design and implement, making them ideal for implementation in countries in transition such as Serbia. Such countries exhibit frequent gaps between global biomedical knowledge and practice in different medical fields (Haines et al., 2004). The gaps include a lack of healthcare strategies in the field of genetic disorders and rare diseases (e.g., strategies for genetic testing, counseling or treatment; Maltese et al., 2017) and lack of data on the prevalence of FXD in Serbia. Such gaps in clinical practice propelled a recent growing interest in FXD in countries in transition such as Serbia. Yet, as noted the above, the prevalence of FXD and the rate of PM in Serbia is unknown. Why is that? Clinicians very rarely order *FMR1* gene testing as “standard of care” for determining the presence of *FMR1* mutations (Budimirovic and Protic, 2016) due to a range of barriers such as poor funding for primary health care and equipment and supplies, inadequate continuing medical education of health care providers, lack of financial resources and legislations, poor quality of the services and waiting lists, and inadequate salaries (Mizik and Karajicic, 2014). Thus, as an initial step, there is a compelling need to study the gap between knowledge and practice of FXD among medical professionals in Serbia. We hypothesize that there is a major lack of knowledge in this area among medical professionals in Serbia. This study also aimed to establish baseline quantitative and qualitative KAP information about the presence of PM and FM of the fragile X gene among medical professionals in Serbia as well as to begin the knowledge dissemination as an initial education step in this field using a survey with an educational-informative function.

## Materials and methods

This cross-sectional prospective study was conducted in Serbia from October 01, 2016 to January 31, 2017 using a survey that also serves an educational-informative function. The study was approved by the Belgrade University School of Medicine (Institutional Review Board (IRB) (No 29/IX-6; September 21, 2016; PI: Budimirovic, co-PI: Protic).

### Sample

The study was conducted on a large nationally representative sample of medical professionals in Serbia. We administered the KAP survey to almost all senior medical students at Belgrade University School of Medicine, Belgrade, Serbia within the first 2 weeks of their sixth academic year. We also surveyed different specialties (general medicine physicians, pediatricians, and gynecologists) among the primary care physicians (“physicians” in the body text; PCPs in tables) from many regions in Serbia, which was obtained during three distinct stages. First, four main regions of Serbia (Belgrade, Vojvodina, Sumadija, and Southern Serbia) were identified according to Nomenclature of Territorial Units for Statistics (NUTS). Seventeen areas from these regions were identified as the main sampling locations. During the next stage, we selected different specialties' departments within primary care centers that could provide examinations for patients with FXD. Psychiatrists and neurologists have not been a part of the primary care system in Serbia. Next, the study participants from selected areas and departments were selected randomly by a random number table. The informed consent of participants was obtained at the beginning of the study. Anonymity of data was assured to all participants. It was emphasized that the collected data would serve exclusively for statistical analysis, and it would be published only in a summary form as a group to establish a baseline of their KAP related to FXD in Serbia.

### Study instrument and measures

The study's instrument was a self-administered 16-item questionnaire of KAP in FXD. This questionnaire could be found as the Supplementary Material. The design of the questionnaire was based on an extensive database search that included MEDLINE/PubMed, Embase, PsycINFO, and Web of Science, originally performed in July of 2016 and re-ran in September of 2016. The search used a combination of the following keywords: “Knowledge, attitude, and practice,” “fragile X,” “*FMR1* gene,” “*FMR1* gene mutation,” and “fragile X-related disorders.” If the data were limited or not available, an additional search included other fields of relevance (e.g., FXTAS, FXPOI, etc.). In addition to the aforementioned systematic searches and the first author's clinical experiences in the FXD field, the questionnaire was developed by consulting a range of relevant literature involving FXS (Hagerman and Hagerman, 2002; Hagerman et al., 2009; Saldarriaga et al., 2014; Budimirovic et al., 2017), FXTAS (Hall et al., 2014; Hagerman and Hagerman, 2015) and FXPOI (Anido et al., 2007). Content validity testing was performed by sending the questionnaire to a panel of three experts to validate the importance and intelligibility of the questionnaire's 16 items before the final version was distributed.

The KAP Questionnaire of FXD was divided into four sections (I–IV):

- *Socio-demographics*. The participants were asked to report their age, gender, specialty, other qualifications (e.g., subspecialty, master, PhD), and total years of clinical experience.
- *Knowledge*. The purpose of this section was to assess knowledge of the *FMR1* gene mutations and FXD in the surveyed cohorts of physicians and students. The questionnaire included 12 items assessing three components:
  - Knowledge self-assessment (SAK, 2/12, see below)
  - Factual knowledge (FK, 5/12) divided into two parts for knowledge of FM (3/5, questions 1–3/5) and premutation (2/5, questions 4–5/5)
  - Knowledge of empirical evidence (EK, 5/12) divided into two parts for *FMR1* gene mutations testing knowledge (2/5) and FXD drug development knowledge (3/5)
    To expand on the aforementioned (i–iii), the SAK component was defined as an evaluation of the participant's own knowledge of the fragile X gene mutations and FXD and included 2 items.
    The FK component refers to the medical facts about the *FMR1* gene mutations and FXD-related information and included 5 true/false questions divided in two subgroups:
    - Three questions for knowledge of FM (scores ranged from 0 to 3)
    - Two questions for premutation, FXTAS and FXPOI knowledge (scores ranged from 0 to 2): “The *FMR1* gene premutation can cause symptoms like those of Parkinson's disease.” and “The *FMR1* gene premutation can cause primary ovarian insufficiency.”
      All questions included “don't know” option, and nominal scale (correct and incorrect/don't know) was provided for the respondents' convenience in disclosing their responses. The points were given for correct answers.
    The EK component consists of 5 true/false questions (points were given for correct answers) assessing information about:
    - The *FMR1* premutation and FM gene testing (2 questions; scores ranged from 0 to 2): “Are you aware of the availability of an early, precise genetic/medical diagnosis of the *FMR1* gene full mutation and/or premutation?” and “Are you aware of the professional organizations' recommendation on the *FMR1* testing in individuals with neurodevelopmental and neurodegenerative disorders?”
    - The FXD drug development knowledge (3 questions; scores ranged from 0 to 3).
    - The FK and the EK components of knowledge questionnaire had educational-informative function.
- *Attitudes toward additional education in the fragile X gene mutations and FXD*. Question (1/16) based on three-point agreement scale (ranging from 1 “Strongly disagree” to 3 “Strongly agree”) was constructed to assess whether the respondents believe that education in the field of FM and premutation of the fragile X gene and FXD such as FXTAS and FXPOI is needed. Thus, assessing respondents' opinions toward educational needs enables us to estimate their opinions on the importance, significance, and severity of *FMR1* gene mutation and FXD.
- *Practice*. This part of the questionnaire consisted of 3 case vignettes (3/16, 2 for PM related FXTAS and FXPOI, respectively) with yes/no follow-up questions, constructed for measuring the competence of physicians and their practice. The vignettes represented hypothetical situations, and respondents were asked whether they would recommend the fragile X gene testing in a certain situation. Very briefly, the case vignette of FXTAS describes a man aged 55 years with a premutation and Parkinson's disease-like symptoms, and the vignette of FXPOI describes a woman aged 45 years with a PM and symptoms of ovarian insufficiency.

### Statistical analysis

Statistical analysis was performed using IBM SPSS Statistics 22 (IBM Corporation, Armonk, NY, USA). Parametric and nonparametric statistics were applied. Results were presented as frequency (percent), median (range), and mean ± sd. Chi-square test was used to test differences between nominal variables (frequencies). Non-parametric Wilcoxon test was used to assess whether there were differences between FK and EK, as well as *FMR1* gene testing and the FXD drug development knowledge in both groups and in total. For ordinal variables, Mann-Whitney *U*-test was used for multiple comparisons. The predictive value of each socio-demographic variable for specific types of knowledge was tested by binary logistic regression and ordinal logistic regression. The confidence intervals for the logistic regression analyses were set at 95%. Multivariate logistic regression models included all variables that were significant in univariate analysis. Variable “specialties” was not included in multivariate analysis because of the multicollinearity with physicians practice setting. For the purpose of this thematic series focusing on premutation, statistical analyses were focused on the interpretation of our study results related to the premutation of the fragile X gene and FXD.

## Results

### Socio-demographic data

All participants who enrolled in the study completed the KAP survey. There were (*n* = 245, 33.5% males) medical students aged 23–30 years and (*n* = 284; 19% males; 176/284, 62.2% in Belgrade) physicians aged 26–64 years in our study. Table 1 depicts the socio-demographic data of the participants (gender and age for both cohorts; practice settings, specializations, other qualifications, and length of clinical experience for the physicians cohort).

**Table 1:** Socio-demographic data in a cohort of PCPs and medical students in Serbia.

**Cohort**	**PCPs (*N* = 284)**	**Medical students (*N* = 245)**
Age (AM ± SD)	48.5 ± 9.87	24.12 ± 0.97
	***N*** **(%)**	***N*** **(%)**
**GENDER**		
Male	53 (18.9)	82 (33.5)
Female	228 (81.1)	163 (66.5)
**PCPs SETTINGS**		
Belgrade	176 (62.2)	
Inner Serbia	107 (37.7)	
**SPECIALIZATION**		
Pediatrics	84 (29.6)	
GP	127 (44.7)	
Gynecology	49 (17.3)	
Without	24 (8.5)	
**QUALIFICATIONS**		
Yes	22 (7.7)	
No	262 (92.3)	
**EXPERIENCE**		
≤ 5 y.	49 (17.3)	
>5 y.	235 (82.7)	

*PCPs, primary care physicians; GP, general practice; Qualifications refers to subspecialty, master's degree, and PhD; Experience refers to total years (length) of clinical experience; y, years*.

*N, number of the study participants; AM, arithmetic mean; SD, standard deviation*.

### Knowledge questionnaire

(i) *Knowledge self-assessment*. Responses to the first question “Have you ever heard about premutation and/or full mutation of *FMR1* gene and fragile X-associated disorders?” revealed that in the physicians group, 176/284 (62%) responders answered “yes” while 229/245 medical students (93.5%) had the same answer (*P* < 0.001). A similar finding for the two groups was also found in assessing their level of FXD knowledge by using a 'knowledge pyramid' (*P* < 0.001). A large percentage of the physicians (117/284, 41.2%) was found to “know nothing” about premutation and FXD whereas roughly half of students reported that they “recognized the basic information” (131/245, 53.5%). Only two physicians (0.7%) and one student (0.4%) thought that they have “professional/expert knowledge” for FXD.

(ii) *Factual knowledge* and (iii) *Knowledge of empirical evidence*. The levels of FK and EK are simultaneously presented and divided in three units in order to allow for easier understanding of the data about FK and EK. The vast majority of these results are presented in Tables 2A, B, and 3, respectively. To illustrate, Tables 2A,B depict data regarding to FK (Table 2A) and EK (Table 2B). In general, the main findings are that: (i) there was a very low level of both types of knowledge in both samples; and that (ii) the medical students had somewhat better overall knowledge than the physicians. The finding was supported by their very low FK very few physicians had all correct answers (Table 2A; 7.5 vs. 3.7% students, *P* < 0.05). As depicted in Tables 2A,B, with respect to all FK and EK questions, the most frequent answers were “don't know” and “no”; these answers were marked more frequently in the physician cohort (ranging from 63.4 to 75.0% for FK and from 73.2 to 91.9% for EK) than in the medical student cohort (ranging from 35.1 to 64.9% and from 40 to 86.9%, respectively). Statistics and details regarding each FK and EK question are presented in Tables 2A,B.

**Table 2A:** Factual Knowledge scores distribution for *FMR1* gene mutations reveals “Don't Know” as the most common answer in a cohort of PCPs and medical students in Serbia.

**Cohort**	**PCPs**	**Medical students**	
**STATEMENT/ANSWERS**			
	***N*** **(%)**	***N*** **(%)**	***P*** **values**
**1. FXS is caused by the** ***FMR1*** **gene FM**.			
Yes	92 (32)	113 (46.1)	
No	12 (4.2)	8 (3.3)	*P* = 0.005
Don't know	180 (63.4)	124 (50.6)	
**2. FXS is the most common cause of inherited ID**.			
Yes	80 (28.2)	91 (37.1)	
No	21 (7.4)	68 (27.8)	*P* < 0.001^*^
Don't know	183 (64.4)	86 (35.1)	
**3. FXS is the most common known single gene cause of autism**.			
Yes	71 (25.0)	101 (41.2)	
No	15 (5.3)	26 (10.6)	*P* < 0.001^*^
Don't know	198 (69.7)	118 (48.2)	
**4. *FMR1* gene PM can cause symptoms like those of Parkinson's disease**.			
Yes	65 (22.9)	74 (30.2)	
No	12 (4.2)	12 (4.9)	*P* = 0.134
Don't know	207 (72.9)	159 (64.9)	
**5**. ***FMR1*** **gene PM can cause primary ovarian insufficiency**.			
Yes	55 (19.4)	70 (28.6)	
No	16 (5.6)	17 (6.9)	*P* = 0.028
Don't know	213 (75.0)	158 (64.5)	

*PCPs, primary care physicians; FXS, fragile X syndrome; FMR1, gene-Fragile X Mental Retardation 1 gene; FM, full mutations; ID, intellectual disability; PM, premutations*.

N, number of the study participants; P values, probability of the data arising by chance;

* *statistically significant data*.

Correct answer is ”Yes.“

**Table 2B:** Empirical evidence knowledge score distribution of *FMR1* gene mutations in a cohort of PCPs and medical students in Serbia.

**Cohort**	**PCPs**	**Medical students**	
	***N*** **(%)**	***N*** **(%)**	***P*****-values**
**Question 1-5: Are you aware of…**			
**Answers:** Yes or No			
**1. …the availability of an early, precise genetic/medical diagnosis of** ***FMR1*** **gene FM and/or PM?**			
Yes	76 (26.8)	147 (60)	
No	208 (73.2)	98 (40)	*P* < 0.001^*^
**2. …the professional organizations' recommendation on** ***FMR1*** **testing in individuals diagnosed with neurodevelopmental and neurodegenerative disorders?**			
Yes	66 (23.2)	91 (37.1)	
No	218 (76.8)	154 (62.9)	*P* < 0.001^*^
			
**3. …advanced phases clinical trials aimed to “translate” new targeted treatments drugs in clinical practice that could modify core problems in FXS related to autism spectrum disorder?**			
Yes	39 (13.7)	43 (17.6)	
No	245 (86.3)	202 (84.2)	*P* = 0.226
			
**4. … the FXS leading the way in clinical trials among all other developmental disorders, including ASD?**			
Yes	23 (8.1)	74 (30.2)	
No	261 (91.9)	204 (83.3)	*P* = 0.002
**5. …0.17 out of 22of these clinical trials aimed to develop targeted drugs focused on the excitatory-inhibitory imbalance in FXS, namely, the mGluR/GABA leading to the excess protein accumulation at dendrite synapses as the hallmark of FXS?**			
Yes	23 (8.1)	32 (13.1)	
No	261 (91.9)	213 (86.9)	*P* = 0.062

*FMR1, gene-Fragile X Mental Retardation 1 gene; PCPs, primary care physicians; FM, full mutations; PM, premutation; ASD, Autism Spectrum Disorder; FXS, fragile X syndrome; mGluR, metabotropic glutamate receptor; GABA, gamma, aminobutyric acid*.

N, number of the study participants; P values, probability of the data arising by chance;

* *statistically significant data*.

**Table 3:** Statistically significant results of univariate and multivariate ordinal and binary logistic regression of PCPs knowledge related to PM, FXTAS, FXPOI and *FMR1* gene testing.

**Univariate and multivariate ordinal logistic regression analysis: dependent variable is premutation factual knowledge score**						
	**Univariate ordinal logistic regression analysis**		**Multivariate ordinal logistic regression analysis**			
Independent variable	OR (95% CI)	*P*	OR (95% CI)	*P*		
Age (*year*)	0.97 (0.94–1.00)	0.027^*^				
PCPs Settings (*BG vs. in. Serbia*)	0.28 (0.17–0.49)	<0.001^*^	0.37 (0.21–0.65)	<0.001^*^		
Experience	0.62 (0.40–0.95)	0.029^*^				
**Univariate and multivariate ordinal logistic regression analysis: dependent variable is knowledge of empirical evidence refers to** ***FMR1*** **gene testing**						
	**Univariate ordinal logistic regression analysis**		**Multivariate ordinal logistic regression analysis**			
Independent variable	OR (95% CI)	*P*	OR (95% CI)	*P*		
PCPs Settings (*BG vs. in. Serbia*)	0.23 (0.14–0.38)	0.000^*^	0.23 (0.14–0.38)	0.000^*^		
Experience	0.61 (0.40–0.92)	0.019^*^	0.62 (0.40–0.95)	0.029^*^		
**Univariate and multivariate binary logistic regression analysis: dependent variable is question “Do you know that** ***FMR1*** **gene premutation can cause symptoms like those of Parkinson's Disease?”(question: II/2/4)**						
	**Univariate binary logistic regression analysis**			**Multivariate binary logistic regression analysis**		
Independent variable	B (SE)	OR (95% CI)	*P*	B (SE)	OR (95% CI)	*P*
PCPs Settings (*BG vs. in. Serbia*)	1.28 (0.29)	3.61 (2.03–6.42)	0.000^*^	1.03 (0.31)	2.81 (1.52–5.20)	0.001^*^
Experience	−0.66(0.23)	0.52 (0.33–0.82)	0.005^*^	−0.71 (0.29)	0.49 (0.28–0.87)	0.015^*^
**Univariate binary logistic regression analysis: dependent variable is question “Do you know that*****FMR1*** **gene premutation can cause primary ovarian insufficiency?” (question: II/2/5)**						
	**Univariate binary logistic regression analysis**					
Independent variable	B (SE)	OR (95% CI)	*P*			
PCPs Settings (*BG vs. in. Serbia*)	1.53 (0.32)	4.64 (2.47–8.71)	0.000^*^			
**Univariate and multivariate binary logistic regression analysis: dependent variable is question “Are you aware of the availability of early, precise genetic/medical diagnosis of** ***FMR1*** **gene mutations?”(qusetion: II/3/1)**						
	**Univariate binary logistic regression analysis**					
Independent variable	B (SE)	OR (95% CI)	*P*			
PCPs Settings (*BG vs. in. Serbia*)	1.58 (0.29)	4.87 (2.77–8.57)	0.000^*^			
Experience	−0.59 (0.23)	0.56 (0.36–0.87)	0.010^*^			
**Univariate binary logistic regression analysis: dependent variable is question “Do you know that there is the professional organizations' recommendation on** ***FMR1*** **gene testing in individuals diagnosed with autism spectrum disorders?” (question: II/3/2)**						
	**Univariate binary logistic regression analysis**					
Independent variable	B (SE)	OR (95% CI)	*P*			
PCPs Settings (*BG vs. in. Serbia*)	1.28 (0.29)	3.61 (2.03–6.42)	0.000^*^			

*Only statistically significant predictors are shown in the table*.

*PCPs, primary care physicians; FMR1, gene-Fragile X Mental Retardation 1 gene; BG, Belgrade; in Serbia, inner Serbia; GP, general practice; Experience refers to total years (length of clinical experience)*.

* *Statistically significant data; P values, probability of the data arising by chance; B, the coefficient for the constant (also called the “intercept”) in the model; SE, the standard error around the coefficient for the constant. OR-an odds ratio as a measure of association between an exposure and an outcome; CI, 95% confidence interval is used to estimate the precision of the OR*.

In order to compare FK of FM/PM and EK of “*FMR1 gene testing”*/“*FXD drug development*” in both cohorts, separate statistical analyses were performed. These results are as follow:

1. FK knowledge of PM
  (1a) The levels of FK of premutation were significantly higher compared to levels of FK of FM among all participants (Wilcoxon test, *z* = −10.90, *P* < 0.05, *r* = −0.34).
  (1b) These results were separately confirmed in both samples.
  (1c) The medical students group was found to have greater FK of premutation, FXTAS, and FXPOI than the physicians group (Mann-Whitney test, *U* = 29967.5; *P* < 0.05).
2. EK of “FMR1 gene testing” and “FXD drug development”
  (2a) The levels of EK of “*FMR1 gene testing”* were significantly higher compared to EK of “*FXD drug development*” among all participants (Wilcoxon test, *z* = −8.11, *P* < 0.05, *r* = −0.25).
  (2b) The same finding was obtained in both samples separately (the physicians group: Wilcoxon test, *z* = −7.42, *P* < 0.05, *r* = −0.31; students group: Wilcoxon test, *z* = −8.02, *P* < 0.05, *r* = −0.32).
  (2c) Medical students showed higher level of EK related to the “*FMR1 gene testing”* than physicians (Mann-Whitney test, *U* = 23,052.5; *P* < 0.05).
  (2d) The students had better EK related to “*FXD drug development*” than the physicians (Mann-Whitney test, *U* = 30639.0; *P* < 0.05).

In the next stage of our statistical analysis, we separately analyzed knowledge of the physicians because independent variables such as practice settings, specializations, other qualifications, and length of clinical experience were missing in the student's cohort.

The FK related to FM and premutation and EK related to “*FMR1* gene testing” and “FXD drug development” in the physicians' cohort was compared according to their gender, clinical practice settings, specialties, and the length of their clinical experiences. The most important findings (Mann-Whitney and Chi-squared tests) are as follow: (i) physicians from inner Serbia had higher level of FK for FM and PM than physicians from Belgrade (*U* = 6255.0; *P* < 0.001 for FK of FM *U* = 7036.5; *P* < 0.001 for FK of premutation), (ii) physicians from inner Serbia had higher level of EK related to the fragile X gene testing than those from Belgrade (*U* = 6186.5; *P* < 0.001), and (iii) physicians with ≤ 5 years of their practice had better FK of premutation, FM (*U* = 4728.5, *P* < 0.05 for FM; and *U* = 4830.0; *P* < 0.05 for premutation), and EK of the fragile X gene testing (*U* = 4718.5; *P* < 0.05) than those with >5 years of clinical practice. There were no statistically significant differences between physicians' knowledge based on other variables such as gender, specialties and other qualifications (data not shown).

To analyze knowledge related to premutation in more detail with regards to FXTAS, FXPOI, and the fragile X gene testing, univariate and multivariate ordinal and binary logistic regression were performed. The following paragraph describes the results.

The results presented above were confirmed by ordinal and/or binary logistic regression (Table 3). Note that only statistically significant predictors are shown in the Table 3. In addition, the main findings of ordinal logistic regression are as follow: (i) physicians practice setting (inner Serbia vs. Belgrade only) predicts FK related to PM of the fragile X gene mutation, FXTAS and FXPOI; (ii) The FK of premutation was significantly higher in physicians with shorter clinical experience (≤ 5 years); (iii) physicians practice setting (inner Serbia vs. Belgrade only) predicts EK of *FMR1* gene testing; (iv) The EK of *FMR1* gene testing was significantly higher in physicians with shorter clinical experience (≤ 5 years). The results of binary logistic regression reveal that the FK of FXTAS was statistically significantly higher in physicians with shorter clinical experience (≤ 5 years), while physicians from inner Serbia and those with shorter clinical experience had higher level of the *FMR1* gene testing. There were no other statistically significant differences between physicians' knowledge related to premutation, FXTAS, FXPOI, and *FMR1* gene testing based on other variables such as gender, specializations and other qualifications (data not shown).

### Attitudes toward additional education in FMR1 gene mutation and FXD

The data indicated that there was a significant difference in attitude toward education in *FMR1* gene mutation and FXD between physicians and medical students (*P* < 0.001). Significantly larger number of physicians (267/284, 94.0%) than students (154/245, 62.9%) reported that they “strongly agree” with the introduction of courses in the field of the fragile X gene mutations. In contrast, only 1 physician (0.4%) and 12 students (4.9% of them) reported that they “strongly disagree” with that kind of education (data not shown).

### Practice questionnaire

Data revealed that the students (229/245, 93.5%) more frequently recommended genetic DNA testing based on hypothetical situations described in three vignettes (2/3 related to premutation) compared to physicians (184/284, 64.8%) (*P* < 0.001). Five (0.9%) respondents (all physicians) stated “it depends on other factors” (data not shown).

## Discussion

To our knowledge, this is the very first KAP study of disorders related to fragile X aimed at assessing awareness of the fragile X gene mutations, specifically *FMR1* gene premutation, and disorders related to these mutations among medical professionals in Serbia and southeast Europe.

In support of our hypothesis, we found low level of knowledge in this genetic/medical field in Serbia. Briefly, senior medical students, physicians from inner Serbia, and young physicians with relatively short clinical experience (≤ 5 years) typically had a higher level of knowledge of the fragile X gene premutation, *FMR1* gene testing, FXD, and drug development in FXD. Interestingly, these medical professionals had better knowledge of FXD related to premutation than to FM. Furthermore, they had a higher level of knowledge of the genetic DNA mutations testing than knowledge of drug development in FXD. Almost all participants of the study clearly indicated that they would need additional education regarding fragile X gene mutations, including premutation and FXD through curricula in the medical schools and/or continued medical educations. Medical professionals in Serbia showed willingness to use *FMR1* gene mutation tests for PM, if these tests were available. Nowadays, FMR1 gene mutation test is not readily available in Serbia because of the expense and this is perhaps one reason that it is not often ordered.

Although respondents in our study showed a better knowledge of premutation- than FM-related FXD, overall, there is low level of knowledge in the field of fragile X gene mutations in Serbia, which is consistent with previous studies in the field (Acharya and Ross, 2009; Kemper and Bailey, 2009; Acharya and Schindler, 2013; Li et al., 2013). While the low level of knowledge in Serbia calls for an action (see below), this finding is not surprising given the fast pace of medical knowledge over the past decades. Namely, an estimated doubling time of the medical knowledge has dramatically increased over the last 70 years: from 50 years in the 1950s, to 7 years in the 1980s, to 3.5 years in 2010 (Densen, 2011). Moreover, in 2020, it is projected to be 0.2 years—just 73 days, suggesting that the level of general medical knowledge increases faster than our ability to assimilate and apply it effectively (Densen, 2011). Nevertheless, fragile X gene premutation knowledge is still important and worthy of attention due to a wide range of medical, cognitive/developmental, neurological and psychiatric disorders found in PM carriers (Hagerman and Hagerman, 2013; Tassone et al., 2014) and the prevalence of PM worldwide- estimated to affect over 20 million individuals (Tassone et al., 2014). The importance is further highlighted by that PM carriers are 10 times more common in general population than individuals with FM (clinically FXS) (Wheeler et al., 2017). For example, Tassone et al. (2014) have championed an effort in bringing together professionals worldwide with different expertise in the premutation and generating publications aimed to disseminate the rapidly accumulating knowledge regarding the PM. Lozano et al. (2014) described a pilot study of copy number variants in PM carriers, suggesting that the *FMR1* genomic studies may represent the first step in understanding the pathophysiological process of the different aforementioned clinical conditions in premutation. Regardless, timely diagnosis of the fragile X gene PM is important to improve symptoms of these patients by implementation of treatment strategies and behavioral interventions (Hagerman et al., 2009; Tassone et al., 2014), and to help them make appropriate reproductive decisions and recognize the potential impact on family members (Lisik, 2017; Wheeler et al., 2017). While physicians' knowledge of the *FMR1* gene premutation is necessary, it is also important to consider the current existence of thousands of other rare genetic disorders (Global, Genes, Allies in Rare Disease, 2015). Thus, it may be unrealistic to expect from these physicians to have a basic knowledge for most of these disorders. These facts also reveal how hard is for physicians to make decisions on what knowledge is important for the field of rare disorders and when such knowledge is needed to deliver optimal clinical care.

The vast majority of included medical professionals in our study responded to all questions in the factual questionnaire with that they “do not know” the details about the fragile X gene premutation and FXD. Identifying the major weaknesses shown in the healthcare professionals' self-assessment presents a first step in increasing awareness of fragile X gene mutations, focusing on PM, and on associated rare diseases among health professionals in Serbia. Medical students and physicians are not familiar with the type of inheritance and phenotypes of FXD. The lack of knowledge in this medical field has negative consequences on the healthcare in Serbia. For example, the above noted delay in diagnosis reduces the possibility of early intervention and medical treatments and/or family support programs (Bailey et al., 2005) as well as increasing burden and stress on the family and patients, who end up seeing multiple providers and be subjected to inappropriate tests during the diagnostic process (Bailey, 2004). In addition, the delayed diagnoses suggest that the premutation carriers do not have critical information about their reproductive risk. For example, 29% of the families with the fragile X gene full-mutation have two children with FXS before the first is diagnosed (Bailey et al., 2009). Thus, knowledge of fragile X gene mutations and FXD represents a cornerstone of successful management of these patients and their families, including in countries such as Serbia (Budimirovic and Protic, 2016).

Senior medical students and young physicians with shorter clinical experience (≤ 5 years) in our study showed a higher level of knowledge of the fragile X gene premutation, the fragile X DNA testing, FXD, and clinical trials in this field than more senior physicians. These encouraging findings could suggest that the importance of fragile X gene mutations and FXD has been recognized during the last decade by medial schools in Serbia. Simply, younger physicians, as well as medical students, have had more opportunities to acquire such knowledge due to their current or recently completed studies. Thus, new generations of physicians may more frequently recommend and order *FMR1* testing in their daily practice if this test were readily available. This will also result in meaningful collaborations between healthcare institutions in Serbia and developed countries such as the U. S., aiming to enhance knowledge and strategies in the FXD field (Budimirovic and Protic, 2016). Inter-professional education programs vary across countries and many academic institutions benefit from implementation of such programs (Herath et al., 2017).

Our study also revealed that, surprisingly, physicians from the Serbian capital Belgrade demonstrated less factual knowledge regarding the fragile X gene premutation than their colleagues from the rest of country. Belgrade's greater reliance on secondary and tertiary healthcare institutions compared to the rest of the country (Jovanovic, 2016) could partially explain such surprising gaps. According to Riley and Wheeler (2017), it is necessary to identify the public health system issues and barriers in the FXD field in order to move future activities in this field. Such activities could be implemented by clinicians, public health professionals, researchers, as well as by individuals with FXD and their families (Riley and Wheeler, 2017). Our observation is that the lack of education and consequent knowledge in this field, as well as healthcare settings, could be the first and the most important barriers during the development of the FXD field in countries in transition such as Serbia. It is reassuring that almost all participants of our study clearly indicated that they would need additional education regarding FXD through curricula in medical school and/or continued medical education. Otherwise, graduate physicians would have insufficient knowledge and skills necessary for early diagnosis and appropriate treatment of FXD. In general, there is a need to enhance the knowledge of *FMR1* testing among physicians and medical students in countries in transition such as Serbia. While a somewhat leading questions-answers approach of the current version of this study's 16-item informative-educational questionnaire may potentially bias survey responses, the robust key finding of the low level of knowledge in the study's large sample of the surveyed medical professionals in Serbia reveals critical knowledge gaps that need to be addressed through education. To that end, we showed that a well-designed survey with educational-informative function would be an excellent first step in the process of knowledge enhancement in the field of FXD. Using such a survey in this study, we disseminated the basic knowledge of *FMR1* gene mutations and FXD among medical professionals who participated in our survey. This could be a useful model to begin education in the FXD field in other countries with similar educational issues. There are no simple and fast solutions to introduce FXD knowledge to medical curricula and continued medical education, as well as daily medical practice. New forms of interactions between policy-makers, funding agencies, researchers, physicians, and other health service providers are needed to improve this knowledge among medical professionals. This study represents the first useful and successful step in such long-term processes. Most importantly, the patients with FXD and their families would benefit.

While this study was conducted in Serbia, its results could be applied to other low and middle-income, developing countries, and countries in transition worldwide, where the gap is more pronounced between scientific and applied knowledge concerning fragile X gene mutations.

## Ethics statement

This study was carried out in accordance with the recommendations of A guide to developing knowledge, attitude and practice surveys, World Health Organization with written informed consent from all subjects. All subjects gave written informed consent in accordance with the Declaration of Helsinki. The protocol was approved by the Institutional Review Board of Belgrade University School of Medicine (No 29/IX-6; PI: Budimirovic, co-PI: Protic; September 21, 2016).

## Author contributions

DB extensively reviewed the data and executed the design of this manuscript, participated in the interpretation of the data, initially drafted the manuscript, modified the tables throughout, critically reviewed and revised the whole manuscript, determining its final content; and wrote the overall conclusions. SC assisted with design of this manuscript, participated in interpretation of the data and helped with development of survey, wrote the subsections of the manuscript and helped finalize them, and critically reviewed and revised the manuscript. ZB assisted with the design of the survey and the manuscript, participated in the process of statistical analysis and interpretation of the data, critically edited the manuscript. PD assisted with design of the article, participated in gathering the reviewed literature, revised one of key subsections of the manuscript, critically reviewed and revised the manuscript. DP initiated the study in Serbia, conducted research, the design of the survey and the manuscript, participated in the interpretation of the data, wrote the subsections and initially drafted the manuscript, together with DB developed and modified the tables throughout, critically revised the manuscript, and coordinated all necessary efforts among coauthors on the manuscript throughout the process.

### Conflict of interest statement

The authors declare that the research was conducted in the absence of any commercial or financial relationships that could be construed as a potential conflict of interest.
