# Supplementary material for: Fragile X-Associated Disorders in Serbia: Baseline Quantitative and Qualitative Survey of Knowledge, Attitudes and Practices Among Medical Professionals
Source: Front Neurosci. 2018 Sep 21;12:652. doi: 10.3389/fnins.2018.00652 (PMC6160902; doi:10.3389/fnins.2018.00652)
Supplement: Supplementary file 1 [file Data_Sheet_1.docx]

Supplementary Material

Fragile X-associated disorders in Serbia: baseline quantitative and qualitative survey of knowledge, attitudes and practices among medical professionals

**Dejan B. Budimirovic*, Smiljana Cvjetkovic, Zoran Bukumiric, Phan Q. Duy, Dragana Protic***

*** Correspondence:** Dejan B. Budimirovic, [dbudimi1@jhu.edu](mailto:dbudimi1@jhu.edu);

Dragana Protic, dragana.protic@med.bg.ac.rs

The survey is anonymous, so please do not enter your name.

**‘Fragile X-associated disorders in Serbia: baseline quantitative and qualitative survey of knowledge, attitudes and practices among medical professionals’**

**The informative-educational survey**

**I) Demographics Questionnaire**

Age: ____ Gender: F / M Specialization: ________________

Other qualifications: ______________________________________________________

(for example: Master, PhD, subspecialization)

Length of clinical experience: 0-1 y 1-5 y ≥ 5 y

**II) Knowledge Questionnaire**

*1. Self-assessment knowledge*

1. Have you ever heard about Fragile X-associated disorders (FXD)?

Yes No

2. If you have ever heard about FXD, please identify the field in the *'knowledge pyramid'*  which is appropriate for your level of knowledge:


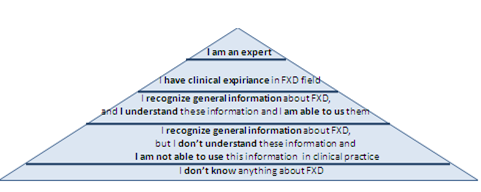


*2. Factual knowledge*

1. Fragile X Syndrome (FXS) is caused by the *FMR1* gene full mutation.

Yes No I do not know

2. FXS is the most common cause of inherited intellectual disability (ID).

Yes No I do not know

3. FXS is the most common known single gene cause of autism.

Yes No I do not know

4. *FMR1* premutation (PM; 55-200 CGG repeats) can cause symptoms like those of Parkinson's Disease (FXTAS: Fragile X Tremor Ataxia Syndrome). FXTAS occurs at ages 50−60 years in approximately 40−50% of male carriers and 16% of female carriers with an average age of onset at 62 years.

Yes No I do not know

5. *FMR1* PM can cause primary ovarian insufficiency (FXPOI: fragile X-associated primary ovarian insufficiency). Around 20% of females with *FMR1* PM may develop FXPOI that manifest with early signs of menopause in the 30s.

Yes No I do not know

*3. Knowledge of empirical evidence*

Are you aware of…:

1. ... the availability of early, precise genetic diagnosis of *FMR1* gene FM and/or PM?

Yes No

2. ... the professional organizations' recommendation on *FMR1* testing in individuals diagnosed with neurodevelopmental and neurodegenerative disorders??

Yes No

3. ...advanced phases clinical trials aimed to ‘translate’ new targeted treatments drugs in clinical practice that could modify core problems in FXS related to autism spectrum disorder?

Yes No

4. …the FXS leading the way in clinical trials among all other developmental disorders, including ASD?

Yes No

5. ...17 out of 22of these clinical trials aimed to develop targeted drugs focused on the excitatory-inhibitory imbalance in FXS, namely, the mGluR/GABA leading to the excess protein accumulation at dendrite synapses as the hallmark of FXS?

Yes No

**III) Attitude**

1. Should education in FXD field be included in continental medical education (CME) and curriculum at undergraduate and postgraduate level of medical studies?

Yes, completely Yes, partially No

**IV) Practice Questionnaire (Three vignettes)**

If you know that it is availability, in your country, of early, precise genetic diagnosis of *FMR1* gene FM and/or PM by blood DNA analysis, and your patient is ...:

1. A child with intellectual developmental disability, such as boy with autism, or shy girl, student in high school, with serious learning problems, especially with problems in mathematics

... Would you refer the patient to the *FMR1* gene testing?

Yes No _____________________________

It depends on other factors (write)

2. A man, aged between 55 y., with neurological disorder with symptoms like those of Parkinson's Disease...

... Would you refer the patient to the *FMR1* gene testing?

Yes No _____________________________

It depends on other factors (write)

3. A women, aged 40 y., with early signs of menopause and infertility?

... Would you refer the patient to the *FMR1* gene testing?

Yes No _____________________________

It depends on other factors (write)
